# Brown adipose tissue and glucose homeostasis – the link between climate change and the global rise in obesity and diabetes

**DOI:** 10.1080/21623945.2018.1551689

**Published:** 2018-12-03

**Authors:** Michael E. Symonds, Grace Farhat, Peter Aldiss, Mark Pope, Helen Budge

**Affiliations:** a Early Life Research Unit, Division of Child Health, Obstetrics & Gynaecology, School of Medicine, University of Nottingham, Nottingham, UK; b Nottingham Digestive Disease Centre and Biomedical Research Centre, School of Medicine, University of Nottingham, Nottingham, UK; c School of Health Sciences, Liverpool Hope University, Hope Park, Liverpool, UK

**Keywords:** brown adipose tissue, glucose, mitochondria

## Abstract

There is increasing evidence that the global rise in temperature is contributing to the onset of diabetes, which could be mediated by a concomitant reduction in brown fat activity. Brown (and beige) fat are characterised as possessing a unique mitochondrial protein uncoupling protein (UCP)1 that when activated can rapidly generate large amounts of heat. Primary environmental stimuli of UCP1 include cold-exposure and diet, leading to increased activity of the sympathetic nervous system and large amounts of lipid and glucose being oxidised by brown fat. The exact contribution remains controversial, although recent studies indicate that the amount of brown and beige fat in adult humans has been greatly underestimated. We therefore review the potential mechanisms by which glucose could be utilised within brown and beige fat in adult humans and the extent to which these are sensitive to temperature and diet. This includes the potential contribution from the peridroplet and cytoplasmic mitochondrial sub-fractions recently identified in brown fat, and whether a proportion of glucose oxidation could be UCP1-independent. It is thus predicted that as new methods are developed to assess glucose metabolism by brown fat, a more accurate determination of the thermogenic and non-thermogenic functions could be feasible in humans.

There is increasing evidence that the rise in diabetes is partly mediated by the increase in global temperatures over the past 20 years.^1,2^ This has been observed across the general population in the USA^3^ and, in pregnant women in Canada relative to the onset of gestational diabetes.^4^ Moreover, the prevalence of gestational diabetes in Canada is higher in the summer and rising ambient temperatures in the 3–4 weeks prior to third trimester glucose tolerance testing can predict gestational diabetes onset.^5^ Consequently as brown fat is highly sensitive to changes in ambient temperature and is normally activated by cold exposure it would be expected to become less active as temperature rises.^6,7^ The unique capacity of brown fat to rapidly respond to cold exposure resides within uncoupling protein (UCP)1 that is located on the inner mitochondrial membrane.^8^ When activated this results in the free flow of protons across the inner mitochondrial membrane,^8^ thereby bypassing the need to convert ADP to ATP, as occurs in the mitochondria of all other tissues.

**Figure 1.**
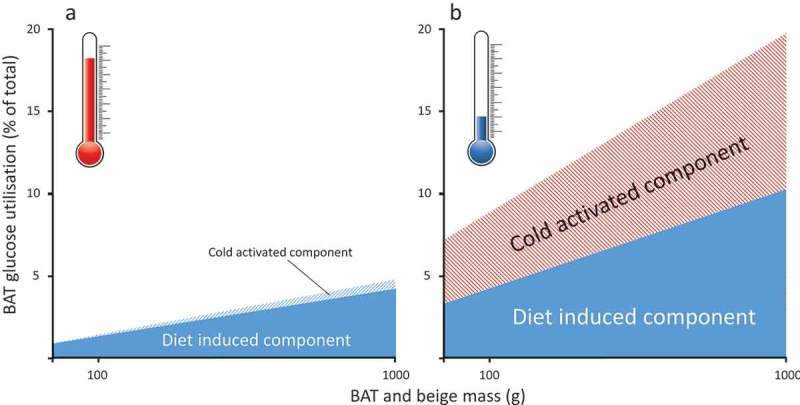
Summary of the potential change in glucose utilisation by brown and beige fat between (A) warm and (B) cool ambient temperature increases. Overall the fraction of whole body-glucose utilisation increases in parallel with an increase in the amount of brown and beige fat, but this is lower in the warm. It is based on calculated estimates of glucose oxidation in adult humans as determined in the cold (e.g.^13^) or after feeding (e.g.^40^).

**Figure 2.**
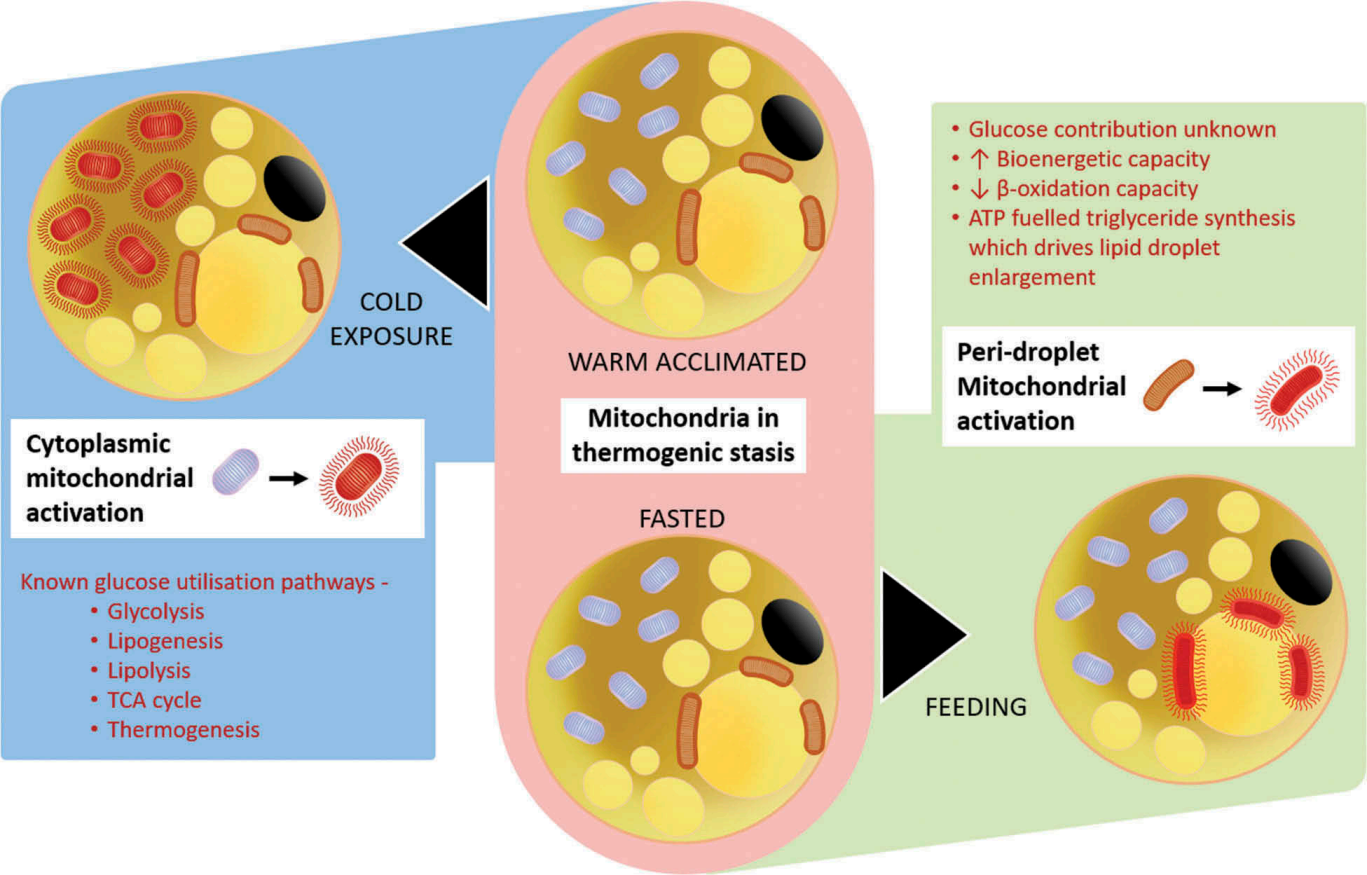
Summary of the potentially different responses between the peridroplet and cytoplasmic mitochondrial fractions within brown (and beige) fat to oxidative metabolism in response to diet or cold-exposure.

The presence of brown fat is adult humans was originally identified from positron emission tomography-computed tomography (PET-CT) studies in cancer patients,^9^ and has been confirmed across a range of ethnicities including Caucasian,^6^ Asian^10^ and African^11^ populations. This technique is dependent on subjects showing an increase in radio-labelled glucose uptake within their brown fat, a response that can be modulated by season and sensitivity to cold.^12^ Consequently the extent to which environmentally induced changes in brown fat function can impact on glucose homeostasis remains a matter of debate.^13^ It should be noted that with repeated PET-CT scans on the same subject then brown fat is identifiable in most, if not all, adults,^14^ and comparable quantification of brown fat has been shown between PET-CT and thermal imaging.^15^ Consequently, it is likely that brown fat is present in all adults,^16^ and as shown in rodents its temperature fluctuates appreciably over a 24h period.^17^ The acute sensitivity of brown fat to changes in temperature would thus mean that an overall rise in current global temperature (see https://climate.nasa.gov/vital-signs/global-temperature/) would be sufficient to reduce its activity on a population wide basis. Moreover, if the United Nations report on climate breakdown (see http://www.ipcc.ch/report/sr15/) is not swiftly acted upon then an even greater challenge would present itself.

## What is the contribution of brown fat to whole body glucose homeostasis?

The primary factors that determine glucose consumption by brown fat are the total amount of fat, its rate of glucose oxidation and capacity to transport glucose.^18^ A number of important recent publications have demonstrated that summary estimates appear to substantially underestimate each of these measures. It is therefore highly likely that current calculations suggesting only 1% of total daily glucose utilisation is partitioned across brown fat are inaccurate.^13^ The total contribution of brown fat should therefore be revised due to the following:

1. The amount of brown fat in adult humans is routinely underestimated, mainly due to the current imaging techniques and the difficulty in measurement because of the mixing of brown and beige fat with other white fat depots in multiple sites in the body.^19^ Beige fat is defined as being a discrete region within white fat that possesses UCP1 although at approximately ten-fold lower concentrations than “classic” brown fat,^20^
2. Brown fat can be activated by diet^21,22^ to the same degree as by cold exposure.^21^ The extent to which these dual activation pathways may be additive is unknown as current studies on cold exposure have been conducted in fasted subjects.
3. Brown fat shows appreciable metabolic activity in warm ambient temperatures, effects that remain for at least two hours after removal of cold exposure.^23^

It is now apparent that the total amount of brown and/or beige fat in adult humans could be up to ten-fold higher, even in obese adults.^19^ This is based on studies that have been able to conduct repeated PET-CT scans of the same individual,^14^ together with further refinements in image analysis.^19^ Furthermore, a significant proportion of adipocytes present in brown or beige depots do not appear to be activated by acute cold exposure. Consequently, we suggest that as much as 20% of daily glucose oxidation could be potentially accounted for within brown fat, as a consequence of either diet and/or cold exposure (see Figure 1). This is in accord with the recognition that brown fat has a regulatory role in glucose homeostasis^18^ explaining why cold-induced stimulation of brown fat has the potential to improve glucose metabolism in both lean^24^ and diabetic^25^ subjects. Indeed, it has recently been shown in obese adults, that long term caloric restriction sufficient to reduce body weight by 16.5% (primarily due to fat loss) promoted the brown adipocyte content in subcutaneous fat by 10%.^26^ At the same time, fasting blood insulin and glucose were improved. Furthermore, in humans, brown fat appears to exhibit a glucose responsive biorhythm that is disrupted when the abundance of brown fat is low.^27^

## Is glucose metabolism by brown fat independent of UCP1 mediated thermogenesis?

Glucose utilisation within UCP1-containing adipocytes in brown and beige fat can occur independently of UCP1-mediated thermogenesis.^18^ This would explain the observation of substantial glucose present in brown fat depots,^28^ and its appreciable utilisation even at thermoneutrality.^23^ Glucose present within brown fat could act, in part, as a reserve to be utilised during cold exposure, as the amount of glucose taken up within supraclavicular brown fat, for example, is closely associated with cold-induced thermogenesis.^28^ Cold exposure is also likely to be accompanied by increased uptake of triglycerides which, in murine obesity models, results in improved glucose homeostasis and up to a five-fold rise in glucose uptake within interscapular brown fat.^29^ If triglyceride uptake is inhibited pharmacologically, then the uptake of glucose by brown fat is greatly reduced whereas, in other tissues such as skeletal muscle, it is unaffected.^30^ Gene deletion studies in mice indicate an increasing number of pathways which can restrict glucose uptake by brown fat.^18^ These appear to be linked to glucose transporter 4, e.g. the GAP complex RalGAP which, when inactivated, results in a seven-fold rise in glucose uptake by brown fat.^31^ It is likely that other pathways are involved and that these may differ between brown and beige adipocytes. For example, deletion of endonuclease G is associated with increased expression of thermogenic genes in beige, but not brown, adipocytes.^32^ This, is turn, is accompanied with improved glucose homeostasis and reduced white fat mass. Indeed, multiple pathways are involved and extend to a wide range of signalling molecules as identified in mice e.g. DJ-1,^33^ although these need confirming in humans.

## Two types of brown fat mitochondria and their differential roles in energy balance

The concept that the regulation of UCP1 differs between brown and beige adipocytes and that the utilisation of glucose by these different cell populations requires further investigation. Glucose oxidation by beige fat has been shown to be independent of UCP1 and is, therefore, non-classical.^34^ The potential divergence in mitochondrial function between dietary and cold-induced thermogenesis could be partly explained by the recent discovery that brown fat contains two different types of mitochondria i.e. the peridroplet and cytoplasmic mitochondrial sub-fractions.^35^ It has been suggested that these fractions are functionally different in their bioenergetic capacity and fatty acid oxidation despite both possessing UCP1. One potential consequence is that there is a greater recruitment of lipid-droplets within the peridroplet mitochondrial domain after feeding,^35^ and perhaps cytoplasmic mitochondria are dominant with cold exposure (see Figure 2). Such an adaptation to feeding would be in accord with the diurnal rhythm in brown fat activity seen in mice, which is consistent with a lower postprandial lipid response, in the morning compared to evening in humans.^36^ The fundamentally different processes between the peridroplet and cytoplasmic mitochondrial sub-fractions^35^ have yet to be examined in different human disease states. These types of investigations could determine whether glucose metabolism differs between each domain. They could also start to explain the recent demonstration of considerable heterogeneity in nutrient, including glucose uptake by brown adipocytes.^37^

## Future research on the role of brown and/or beige adipocytes on glucose homeostasis

Given the increasing evidence that brown and/or beige fat has a role in both dietary and cold-induced thermogenesis, more focus is now required on the impact of diet especially under thermoneutral conditions.^38^ A combined effect of diet and cold exposure could therefore herald ground-breaking approaches to diabetes prevention and/or treatment. The urgent need to make such an intervention is high-lighted by the continued rise in global temperatures, and the increased duration of “summer” (see https://www.metoffice.gov.uk/binaries/content/assets/mohippo/pdf/uk-climate/state-of-the-uk-climate/soc_supplement-002.pdf) which currently appear to be largely unpreventable. Moreover, the impact of ageing needs to be considered as this is accompanied with a “natural” decline in brown fat mass, which could underpin the onset of type 2 diabetes.^39^ Critically, more sophisticated assessments (including the potential use of glucose tracers) to accurately assess glucose uptake by brown adipose tissue and of UCP1, both in vivo and in vitro, are required to enable a more accurate partitioning of its thermogenic and non-thermogenic functions.
